# First application of a protein-based approach for time since death estimation

**DOI:** 10.1007/s00414-016-1459-4

**Published:** 2016-10-21

**Authors:** Stefan Pittner, Bianca Ehrenfellner, Angela Zissler, Victoria Racher, Wolfgang Trutschnig, Arne C. Bathke, Alexandra M. Sänger, Walter Stoiber, Peter Steinbacher, Fabio C. Monticelli

**Affiliations:** 1 Department of Cell Biology and Physiology, University of Salzburg, Hellbrunnerstr. 34, 5020 Salzburg, Austria; 2 Department of Mathematics, University of Salzburg, Hellbrunnerstr. 34, 5020 Salzburg, Austria; 3 Department of Forensic Medicine and Forensic Neuropsychiatry, University of Salzburg, Ignaz-Harrer-Straße 79, 5020 Salzburg, Austria

**Keywords:** Time of death, Postmortem interval (PMI), Method, Muscle, Protein, Degradation, Case

## Abstract

Awareness of postmortem degradation processes in a human body is fundamental to develop methods for forensic time since death estimation (TDE). Currently, applied approaches are all more or less limited to certain postmortem phases, or have restrictions on behalf of circumstances of death. Novel techniques, however, rarely exceed basic research phases due to various reasons. We report the first application of a novel method, based on decay of muscle proteins, in a recent case of murder-suicide, where other TDE methods failed to obtain data. We detected considerably different protein degradation profiles in both individuals involved and compared the data to our presently available database. We obtained statistical evidence for un-simultaneous death and therefore received valuable information to trace the progression of events based on protein degradation. Although we could not sensibly convert the data to respective times of death, this case highlights the potential for future application and elucidates the necessary further steps to develop a viable TDE method.

## Introduction

Time since death estimation (TDE) is a central task in forensic sciences. However, the few reliable methods available to date all suffer from notable applicability restrictions. The temperature method [1, 2], the most precise and most frequently used method, can only be used until ∼36 h postmortem (hpm) depending on individual and environmental conditions. Furthermore, the applicability in certain circumstances of death such as burning or massive trauma can be doubted. Other postmortem changes, used for the delimitation of the postmortem interval (PMI) such as the development of *rigor* and *livor mortis*, are also restricted to specific circumstances. These especially include no major blood loss or heavy trauma [3]. Furthermore, the informative gain of the aforementioned methods is generally limited to the first 1 or 2 days postmortem [4]. Viable information in later phases, by means of a minimum PMI (PMI_min_), can be obtained by forensic entomology. This approach, however, is generally restricted to local fauna and respective insect accessibility to the dead body [5].

Additional problems arise under specific environmental conditions, or circumstances of death, such as in cases where the remains are detected in, or under water. Water has a specific set of properties that are known to have high influence on *postmortem* changes. Due to the higher thermal conductivity of water, especially temperature has an even greater impact, than on land and accounts, for example, for a faster adjustment of body temperature [6]. Water-submerged corpses undergo well-defined morphological changes. These are, however, of no additional benefit for PMI estimation in cases combining low water temperatures and submersion for relatively limited time. As depicted in tables listing morphologic changes in relation to minimum postmortem submersion interval (PMSI), signs of advanced decomposition (e.g., nail loss, brain liquefaction) may already be present after 3 days postmortem under warm conditions (e.g., in 19 °C water in summer), whereas under cold conditions (e.g., in 3.5 °C water in winter), the earliest morphologic changes apart from wrinkling (e.g., marbling, peeling) of the skin do not even regularly occur until 30 days [6–8]. Such morphology-based estimations have to be treated with additional caution, as they are afflicted with limitations, for example in later postmortem phases [9] and in sequestered aquatic environments [10].

Reliable information on the time since death are often gathered from non-biomedical sources such as photographs or videos taken from traffic or public place surveillance, traces including public transport tickets found at the scene, testimonies, or confessions [4]. These clues, however, are often susceptible to forgery or based on more or less defined assumptions.

There is no doubt about the need for additional, reliable TDE techniques. Forensic science departments around the world take great research effort into this field. Promising results for future methods were achieved in animal models, focusing on analyses of RNA degradation and colorimetric changes of tooth pulp [11] or alterations of the microbial fauna [12, 13]. However, newly discovered biomedical approaches rarely exceed basic research phases for several reasons. For once, many methods are never tested on humans due to the mere unavailability of valid sample material. Additionally, most discovered changes are of a gradual character and thus exclusively provide information when compared to a baseline value. Despite the feasibility to define such a value in a standardized animal model, this is generally impossible in actual forensic cases [4].

We recently reported a muscle protein degradation-based approach to delimitate the time since death in the animal model [14] and verified it as a promising approach in human muscle tissue from actual forensic cases [15]. Although there is still a lot of research required to refer to it as a “method to determine the time since death,” we here report first valuable application of muscle protein degradation analysis in forensic investigations.

Two individuals in a case of murder-suicide, in which other TDE methods failed to deliver results, were analyzed on behalf of skeletal muscle protein degradation and compared on the basis of existing data from human postmortem muscle samples.

## Case

### Finding of the corpses

Two dead bodies were discovered in a lake (water temperature: approx. 4 °C) in Austria. The dismembered corpse of a 72-year-old woman was deposited at the waterside, torso and limbs were separately stored in large suitcases, and the head was casted into a concrete block. During recovery, police divers found the second corpse of a 73-year-old man in close vicinity, at the bottom of the lake. Bags filled with stones were tied around his wrists. At the scene, none of the available TDE methods could be applied for the female. The body temperature was adjusted to environmental temperature, there was no applicable development of *rigor* and *livor mortis*, and there was no macroscopic colonization of necrotrophic organisms. Likewise, the male’s corpse was adjusted to ambient temperature. However, strong, complete *rigor mortis* as well as distinctive lividity, completely disappearing on thumb pressure, was developed. Except wrinkling of the skin in the male, both corpses did not exhibit any water-induced morphologic changes enabling minimum PMSI determination.

### Autopsy

The woman’s torso showed complete proximal postmortem separation of all limbs as well as separation of the head at the third cervical vertebra. The neck region showed significant ligature markings and hemorrhages in neck muscles. Skin in direct contact with the concrete was slightly corroded and depicted no signs of tissue bleeding. Retroauricular and frontal petechiae, however, were consistent with strangulation as cause of death. Note that these areas were covered by the auricles and a tuft of hair, respectively, and were thus unaffected by the concrete.

For the male corpse, drowning was confirmed as cause of death. No major additional injuries were observed.

In both cases, autopsy discovered alike content of the stomach, similarly digested. Hence, from a medical examiner’s point of view, there was no prospect to trace the progression of events. However, there was a weak indication for a more or less simultaneous death, due to similar morphology and equal stomach content.

### Further investigations

Non-biomedical evidence suggested domestic violence in a married couple. Findings in the couple’s house indicated that the husband strangled his wife with a garrote, dismembered her body in the living room, and packed her into suitcases. After a 6+-h drive in his car, he arrived at the scene and deposited the suitcases in the lake. He was identified as a hotel guest who had checked in in person at a certain date. A taxi driver confirmed to have driven him back to the scene 3 days later. This was the night before the finding and autopsy.

The slightly inconsistent findings, 3+ days between the two deaths, and yet evidence for simultaneous death, gave occasion to investigate, whether our novel approach for time since death estimation would reveal biomedical differences of the degree of degradation between the two cases, enhancing one of two possibilities of the progression of events.

### Analysis of muscle protein degradation

**Fig. 1:**
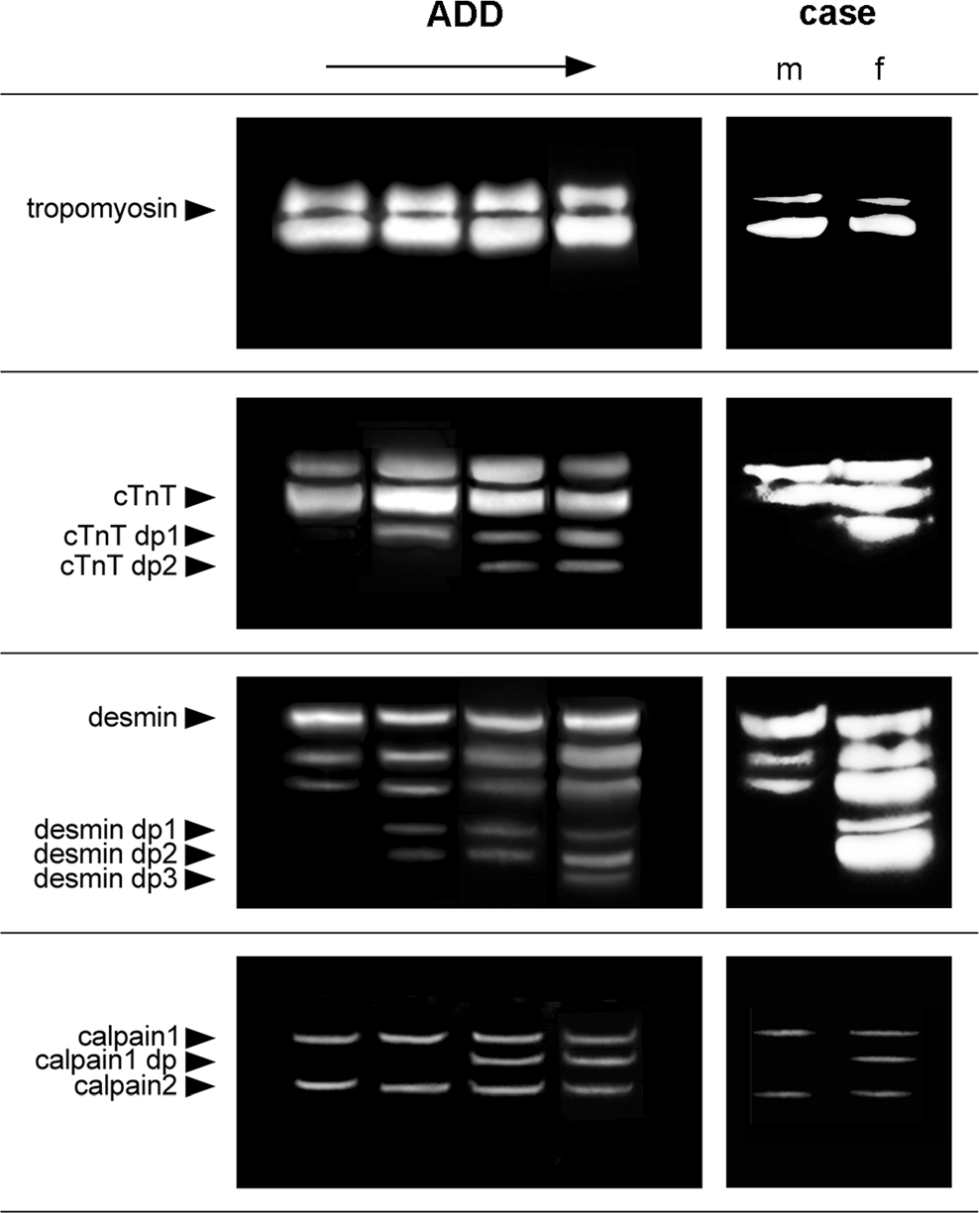
Degradation profiles of selected muscle proteins at increasing ADD (*left column*, images rearranged and adapted from Pittner 2016 [15]) and results of the protein analysis in the present case (*right*, *m* male; *f* female corpse). Notably, the woman’s protein patterns in cTnT, desmin, and calpain resemble advanced stages of decomposition compared to those of the man

Pieces of muscle tissue were removed from the lateral thigh muscles of both individuals and processed according to our standard protocol [15]. Analysis revealed that the degradation profiles of the two individuals differed substantially in three of the four muscle proteins tested. While desmin, cardiac troponin T (cTnT), and calpain analyses of the male corpse resulted in a “native protein pattern,” characteristic degradation products were detected in all three experiments when analyzing the woman’s muscle tissue (Fig. 1). In contrast, tropomyosin, a protein that is known to remain unaffected in early degradation phases and thus aids as a valuable negative control, appeared in comparable fashion in both cases.

In our previous work, we described the dependence of muscle protein degradation upon accumulated degree days (ADD [°*d*] = time [*d*] × temperature [°C]), a measure of energy affecting a system. We worked with logistic regression models reflecting the fact that the probability of observing degradation products increases if ADD increases. Starting with a uniform non-informative prior, applying the Bayes’ Formula and calculating 2.5 and 97.5 % quantiles (i.e., 95 % confidence intervals) as well as the mean of the posterior distribution (separately for both individuals), yield the results presented in Table 1.

**Table 1:** Estimation of ADD and the 95 % confidence interval (CI) in the male (m) and female (f) based on analysis of muscle protein degradation. Δ ADD depicts the difference in the estimated values, as well as the statistical minimum difference between both cases

	Estimated ADD [°d]	CI (95 %)	
		−	+
m	3.3	0.0	7.9
f	25.4	12.1	36.0
Δ ADD	22.1	Min 4.2	

The estimated ADD for both individuals (based on the aforementioned logistic model and 51 earlier cases) differs by 22.1 °d. Moreover, the ADD of the female individual is at least 4.2 °d above the male’s ADD at a probability of 90 %, providing statistic evidence for the succession of the crime. The difference in confidence levels (95 % for individual CIs, 90 % in interindividual comparison) is explained by the fact that each of the 95 % CIs leaves a residual possibility of 5 % on either side (i.e., up or down), so that, in worst case, 90 % confidence remain.

Dividing ADD values by mean environmental temperature theoretically yields the PMI. However, for the reasons discussed below, we refrain from this calculation in the present case.

## Discussion

Here, we report the first application of a protein degradation-based method to trace back the progression of events in a case of murder-suicide.

At the scene and the autopsy room, both individuals showed similar morphology on behalf of postmortem changes that aid PMI estimation. As in many other cases, it was impossible to delimitate the time since death by biomedical evidence. Using biochemical analysis of muscle proteins, we, however, detected remarkable dissimilarities in muscle protein degradation and thus different decomposition states.

TDE approaches have to take into account individual as well as environmental impacts. Especially ambient temperature, widely characterized as the most important influencing factor of degradation processes [4], inevitably has to be considered in both, the employment of data bases and all consequent statistics as well as in each individually investigated case. The concept of ADD is widely accepted, as a feasible approximation. It is understood as a measure of energy obtained by a system (i.e., dead body) and has been shown to correlate with the advance of certain metabolic events (i.e., decomposition) [16–18]. A special relevance of the correlation between postmortem change and ADD has been shown in regard to the estimation of the PMSI [7, 19].

Back calculation to the time since death using the ADDs acquired via protein degradation would be very vague in the present case and is therefore refrained. For once, the underlying model includes only 51 cases, none of them a case of drowning. Additional research is needed to provide evidence on that behalf. Note that Henssge et al. (2000) describe a correction factor of 0.35–0.5 for temperature-based TDE due to water as surrounding medium [1]. Since protein degradation, as any metabolic process is highly dependent upon (body-)temperature, a faster cooling process could heavily decelerate decay. However, in the present case, the focus was not solely on time since death, but on differences between the two individuals. Since they were located in the same environment, at least within the PMI of the male, all according influences cancel out and ADD can thus be interpreted as a direct measure of the degree of decomposition.

With 95 % confidence intervals of almost 8 °d for the man, and over 20 °d for the woman, the informative value based on protein degradation itself is yet somewhat limited. Additional cases in the database and a larger catalog of target proteins will improve the validity of this method significantly. Still, in combination with other TDE methods, or non-biomedical crime scene evidence, the possibility to exclude certain time windows by statistical significance, even now, offers unique opportunities for PMI delimitation.

Without a doubt, intensive research on influencing factors, such as environmental temperature, humidity, age, BMI, and many more, is unconditionally needed until the technique can withstand as a valid method for time since death estimation. Nevertheless, in cases with specific issues that are not in conflict with the current weaknesses, we could prove that this approach already is a valuable tool for TDE-related investigations.

## Conclusion

In many forensic cases, the estimation of the time since death is a very important indicatory aspect for investigations. Witnesses’ testimonies, data gathered from surveillance systems, public transport tickets, or confessions by the perpetrator often deliver valuable information about the progression of events in a criminal act. Due to the possible impact of individual bias or even forgery, however, these data have to be treated with caution and often act merely as indications than as a clear proof.

Time since death estimation methods, based on biomedical changes, to the extent they can be applied, can deliver unforgeable information on the degree of decomposition, and thus provide reliable evidence. The existing methods, however, are often limited to environmental factors or circumstances of death. Hence, the requirement for additional approaches to support the current array is undoubted.

Postmortem muscle protein degradation can be a valuable addition to the current spectrum of time since death estimation methods in the near future. Although there is still a lot of research to be done to define a valuable standard model that involves exclusion criteria, or correction factors for case individual circumstances, we were able to attain relevant information from protein degradation analysis and confirm the progression of events in a criminal case, in which other biomedical methods for time since death estimation failed.

## References

[1] Henssge C, Althaus L, Bolt J (2000). Experiences with a compound method for estimating the time since death. I. Rectal temperature nomogram for time since death. Int J Legal Med.

[2] Henssge C, Althaus L, Bolt J (2000). Experiences with a compound method for estimating the time since death. II. Integration of non-temperature-based methods. Int J Legal Med.

[3] Mathur A, Agrawal YK (2011). An overview of methods used for estimation of time since death. Aust J Forensic Sci.

[4] Madea B (2016) Methods for determining time of death. Forensic Sci Med Pathol 1–35. doi: 10.1007/s12024-016-9776-y 10.1007/s12024-016-9776-y 27259559

[5] Amendt J, Richards CS, Campobasso CP (2011). Forensic entomology: applications and limitations. Forensic Sci Med Pathol.

[6] Reh D med H (1967) Anhaltspunkte für die Bestimmung der Wasserzeit. Dtsch Z Für Gesamte Gerichtl Med 59:235–245. doi: 10.1007/BF00576900 5589868

[7] Heaton V, Lagden A, Moffatt C, Simmons T (2010). Predicting the Postmortem Submersion Interval for Human Remains Recovered from U.K. Waterways*. J Forensic Sci.

[8] Madea B, Doberentz E (2010) Commentary on: Heaton V, Lagden A, Moffatt C, Simmons T. Predicting the postmortem submersion interval for human remains recovered from U.K. waterways. J Forensic Sci 2010;55(2):302-7. J Forensic Sci 55:1666–1667; author reply 1668. doi: 10.1111/j.1556-4029.2010.01517.x 10.1111/j.1556-4029.2010.01517.x 21044068

[9] Introna F, Di Vella G, Campobasso CP (2013). Migrant deaths and the Kater Radez I wreck: from recovery of the relict to marine taphonomic findings and identification of the victims. Int J Legal Med.

[10] De Donno A, Campobasso CP, Santoro V (2014). Bodies in sequestered and non-sequestered aquatic environments: a comparative taphonomic study using decompositional scoring system. Sci Justice J Forensic Sci Soc.

[11] Young ST, Wells JD, Hobbs GR, Bishop CP (2013) Estimating postmortem interval using RNA degradation and morphological changes in tooth pulp. Forensic Sci Int 229:163.e1-6. doi: 10.1016/j.forsciint.2013.03.035 10.1016/j.forsciint.2013.03.035 23647867

[12] Metcalf JL, Wegener Parfrey L, Gonzalez A (2013). A microbial clock provides an accurate estimate of the postmortem interval in a mouse model system. eLife.

[13] Hauther KA, Cobaugh KL, Jantz LM (2015). Estimating Time Since Death from Postmortem Human Gut Microbial Communities. J Forensic Sci.

[14] Pittner S, Monticelli FC, Pfisterer A, et al (2015) Postmortem degradation of skeletal muscle proteins: a novel approach to determine the time since death. Int J Legal Med 1–11. doi: 10.1007/s00414-015-1210-6 10.1007/s00414-015-1210-6 26041514

[15] Pittner S, Ehrenfellner B, Monticelli FC, et al (2016) Postmortem muscle protein degradation in humans as a tool for PMI delimitation. Int J Legal Med. doi: 10.1007/s00414-016-1349-9 10.1007/s00414-016-1349-9 PMC5055573 26951243

[16] Megyesi MS, Nawrocki SP, Haskell NH (2005). Using accumulated degree-days to estimate the postmortem interval from decomposed human remains. J Forensic Sci.

[17] Larkin B, Iaschi S, Dadour I, Tay GK (2009). Using accumulated degree-days to estimate postmortem interval from the DNA yield of porcine skeletal muscle. Forensic Sci Med Pathol.

[18] Lecheta MC, Thyssen PJ, Moura MO (2015). The effect of temperature on development of Sarconesia chlorogaster, a blowfly of forensic importance. Forensic Sci Med Pathol.

[19] Humphreys MK, Panacek E, Green W, Albers E (2013). Comparison of Protocols for Measuring and Calculating Postmortem Submersion Intervals for Human Analogs in Fresh Water. J Forensic Sci.

